# The expansion of liquid biopsies to vascular care: an overview of existing principles, techniques and potential applications to vascular malformation diagnostics

**DOI:** 10.3389/fgene.2024.1348096

**Published:** 2024-01-18

**Authors:** Ann Mansur, Ivan Radovanovic

**Affiliations:** ^1^ Division of Neurosurgery, Department of Surgery, Faculty of Medicine, University of Toronto, Toronto, ON, Canada; ^2^ Department of Laboratory Medicine and Pathobiology, School of Graduate Studies, University of Toronto, Toronto, ON, Canada; ^3^ Division of Neurosurgery, Department of Surgery, Toronto Western Hospital, University Health Network, Toronto, ON, Canada; ^4^ Krembil Brain Institute, University Health Network, Toronto, ON, Canada

**Keywords:** liquid biopsy, vascular malformations, targeted therapy, cfDNA, CTCs, exosomes, sequencing

## Abstract

Vascular malformations are congenital lesions that occur due to mutations in major cellular signalling pathways which govern angiogenesis, cell proliferation, motility, and cell death. These pathways have been widely studied in oncology and are substrates for various small molecule inhibitors. Given their common molecular biology, there is now a potential to repurpose these cancer drugs for vascular malformation care; however, a molecular diagnosis is required in order to tailour specific drugs to the individual patient’s mutational profile. Liquid biopsies (LBs), emerging as a transformative tool in the field of oncology, hold significant promise in this feat. This paper explores the principles and technologies underlying LBs and evaluates their potential to revolutionize the management of vascular malformations. The review begins by delineating the fundamental principles of LBs, focusing on the detection and analysis of circulating biomarkers such as cell-free DNA, circulating tumor cells, and extracellular vesicles. Subsequently, an in-depth analysis of the technological advancements driving LB platforms is presented. Lastly, the paper highlights the current state of research in applying LBs to various vascular malformations, and uses the aforementioned principles and techniques to conceptualize a liquid biopsy framework that is unique to vascular malformation research and clinical care.

## 1 Introduction

Vascular malformations (VMs) are anomalies of vascular development that include venous, lymphatic, arterial, capillary and mixed malformations (Wassef et al., 2015; Kunimoto et al., 2022). They usually affect children and young adults and present with rapid overgrowth and hemorrhage, causing patients significant, pain, deformity, organ failure and functional deficits (McCafferty, 2015; Wassef et al., 2015; Quiesser et al., 2018; Mansur and Radovanovic, 2023). Conventional treatments include surgery, endovascular embolization and/or radiosurgery; however, they are often insufficient in completely treating many patients with complex VMs, resulting in significant morbidity and/or mortality (Gross and Du, 2013; Derdeyn et al., 2017; Mansur et al., 2021; Mansur and Radovanovic, 2023).

Faced with this challenge, clinician-scientists and biologists recognized the need to establish the molecular and genetic underpinnings of these diseases in order to develop targeted therapeutics for this patient population. Over the past 2 decades, significant efforts in this pursuit led to seminal discoveries on the specific mutations that drive each type of vascular malformation and the opportunity to repurpose cancer drugs to target these specific mutations in a novel precision-medicine approach (Couto et al., 2017; Nikolaev et al., 2018; Hong et al., 2019; Fish et al., 2020; Van Damme et al., 2020; Pan et al., 2021; Quiesser et al., 2021; Mansur and Radovanovic, 2023). Hence, it is important to be able to recapitulate a patient’s individual mutational burden to inform these therapeutic decisions in VM care. The current standard of care for establishing a molecular diagnosis in VMs necessitates histopathological evaluation of open biopsy specimens. This is an invasive procedure that carries the risk of bleeding, infection, pain and inconclusive results (Di Sario et al., 2023; Raufi et al., 2023). Also, it may not be suitable when VMs are difficult to access, such as some VMs of the central nervous system.

Liquid biopsy (LB) techniques have revolutionized the field of medical diagnostics and personalized medicine in their ability to efficiently profile genomes and inform therapeutic decisions in a minimally invasive manner, making them attractive alternatives to open biopsies. The recent developments in sequencing techniques enabled more sensitive and cost-effective tests, thereby facilitating its adoption into clinical practice. This is particularly the case in oncology, where there is not only an abundance of literature on the principles and application of LB, but also various commercially available molecular assays that are being increasingly adopted into clinical use (Adashek et al., 2021; Caputo et al., 2023; Raufi et al., 2023). VMs share many of the same underlying mutations as some cancers and given the potential to repurpose cancer drugs for these vascular conditions, broadening the application of LBs to vascular disorders becomes a necessity.

In this review, we explore the principles and analytic tools for LB applied across oncological and non-oncological pathologies, and their developing role in the field of vascular malformations.

## 2 Principles and technologies

**FIGURE 1:**
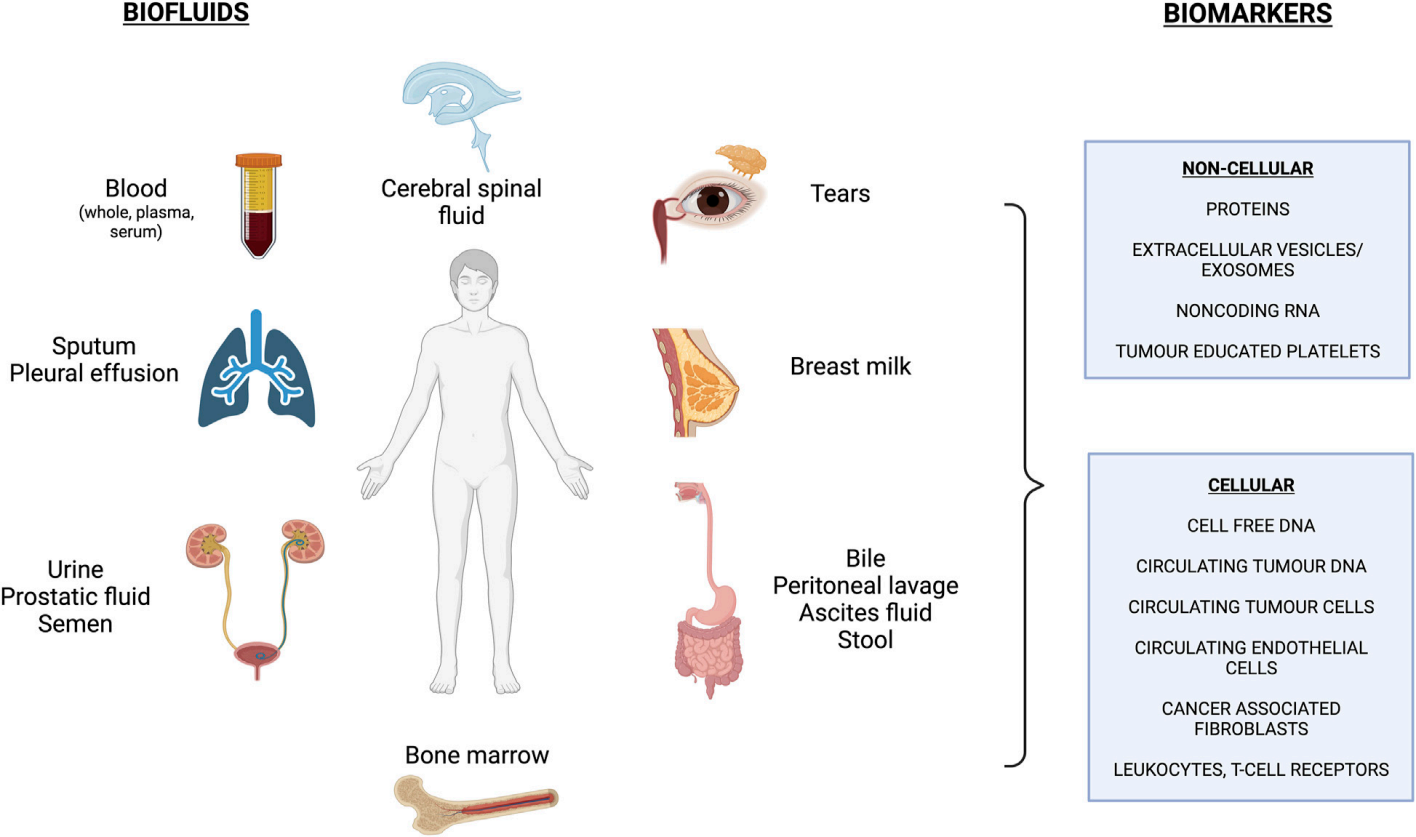
Overview of commonly used biofluids and biomarkers assessed in liquid biopsy applications. *Figure made with BioRender.com (Publication License #UG260GTFD9).

LB involves analyzing biological fluids in order to obtain information on disease-related biomarkers. Figure 1 outlines the array of biofluids that can be interrogated, with blood being the most common one to date. With these samples, we can detect and analyze various circulating biomolecules that are released by healthy cells (cell-free DNA: cfDNA) and by diseased cells such as the “tumour circulome”: circulating tumour DNA (ctDNA), circulating tumour cells (CTCs), exosomes/extracellular vesicles (EVs), tumour educated platelets (TEPs) and circulating tumour proteins and metabolites (Nikanjam et al., 2022; Caputo et al., 2023) (Figure 1). Here we summarize some of these components and their methods of isolation.

### 2.1 cfDNA/ctDNA

cfDNA are small fragments of extracellular DNA (140–200 base pairs long) that are released by either non-tumour (cfDNA) or tumour cells (ctDNA) through apoptosis, necrosis and active secretion processes (Stroun et al., 2000). cfDNA has a half-life of 15 min to 2.5 h before they are cleared by macrophages hepatically (Nikanjam et al., 2022). They can be found in the serum at concentrations of 0–1,000 ng/mL (Siravegna and Bardelli, 2014) but plasma yields a higher quality sample due to less leukocyte contamination (Raufi et al., 2023). In healthy individuals, cfDNA levels are typically between 0 and 100 ng/mL with an average being below 30 ng/mL (Esposito et al., 2017). They arise mostly from hematopoietic cells, and their concentrations correlate with age due to decreased clearance (Moss et al., 2018).

The proportion of cfDNA that is mutant ctDNA is called the variant allele frequency (VAF) and varies with tumour type, location and stage (Bielo et al., 2023). For example, both the concentration and length of the ctDNA have been shown to correlate with the presence of metastatic disease, with the VAF rising from <1% in early stage up to 80% in metastatic disease (Diaz and Bardelli, 2014; Zill et al., 2018).

cfDNA/ctDNA are the most commonly investigated and reported biomarkers in oncology as they can provide information such as the presence of point mutations, copy number variations (CNV), gene fusions, microsatellite instability and methylation changes (Bohers et al., 2021; Wu and Chu, 2022). The rapid evolution of genetic sequencing technologies and assays enabled the expansion of LBs from a strictly academic enterprise to one being adopted into clinical practice. Broadly speaking, the analytical tools lie in the polymerase-chain-rection (PCR) methods, the next-generation sequencing (NGS) techniques or the epigenetic analyses. Each have their own strengths and limitations, and can be carefully applied for specific needs in personalized medicine.

#### 2.1.1 PCR applications

PCR is a sensitive and widely used molecular test in research and clinical laboratories. Table 1 summarizes the types of PCR used for LBs; they all use primers and DNA amplification to detect a predetermined gene of interest. Briefly, real-time quantitative PCR (real time qPCR) is a fast and low-cost approach that measures fluorescent emission of labelled probes to look for a small number of targeted variants with a VAF of 0.1%–10% (Sorber et al., 2017). Digital PCR (dPCR) runs multiple parallel PCR in compartments based on a number of DNA strands, which decreases background noise and improves the accuracy of the test compared to real-time PCR. It is very sensitive and can detect a mutant allele frequency less than 0.1%, but is more expensive than qPCR (Kinde et al., 2011). Droplet dPCR (ddPCR) uses emulsion droplets to separate DNA and can detect up to 5 target mutations in multiple genes at the same time with a VAF as low as 0.01%, making it faster, more efficient and cost-effective if run for multiple patients at the same time (Heitzer et al., 2019; Caputo et al., 2023). It not only detects point mutations, but can also be used to interrogate CNVs and translocations, which are important in the detection of certain cancers such as lymphomas (Bohers et al., 2021). BEAMing (beads, emulsion, amplification and magnetics) combines PCR with flow cytometry to detect known mutations in solid tumours with a VAF as low as 0.01% (Dressman et al., 2003; Diehl et al., 2008). Lastly, techniques such as SERS (surface-enhanced Raman spectroscopy) and UltraSEEK combine PCR with mass spectrometry to harness the power of multiplexing in order to detect mutations with low frequencies in precious samples with low input cfDNA (Pyrak et al., 2019; Lamy et al., 2020).

**TABLE 1:** Comparison of PCR and NGS techniques for isolated cfDNA/ctDNA.

Analysis type	Technique	Target	Sensitivity (%)	References
PCR	Rtq-PCR	Hotspot mutation	0.1–10	Sorber et al. (2017)
	dPCR	Hotspot mutations, fusions, translocations, CNV	0.1	Kinde et al. (2011)
	ddPCR		0.01–0.1	Heitzer et al. (2019), Bohers et al. (2021)
	BEAMing		0.01–0.1	Dressman et al. (2003), Diehl et al. (2008)
NGS	CAPP-Seq	Mutations (known and unknown), CNV, indels, chromosomal rearrangements	0.02	Newman et al. (2014)
	Tam-Seq		2	Gale et al. (2018)
	eTam-Seq		0.02	Caputo et al. (2023)
	Safe-Seq		0.01–0.05	Kinde et al. (2011)
	TEC-Seq		0.05–0.1	Bohers et al. (2021)
	WES	Coding regions, promoters, untranslated regions	5	Van Dijk et al. (2014)
	WGS	Genome-wide structural variants	5–10	Caputo et al. (2023)

Abbreviations: PCR, polymerase chain reaction; rtqPCR, real time quantitative PCR; dPCR, digital PCR; ddPCR, digital droplet PCR; BEAMing, beads emulsion amplification magnetics; NGS, next-generation sequencing; CAPP-Seq, Cancer personalized profiling by deep sequencing; Tam-Seq, Tagged amplicon deep sequencing; TEC-Seq, targeted error correction; WES, whole exosome sequencing; WGS, whole genome sequencing; CNV, copy number variations; indels-insertions and deletions.

#### 2.1.2 NGS applications

PCR is sensitive but is used to investigate known mutations in known genes. NGS, while more costly than PCR applications, can detect both known and unknown mutations covering entire genes or coding regions with a VAF similar to ddPCR, but with the advantages of performing a broader investigation (Raufi et al., 2023). NGS allows deep targeted sequencing and untargeted approaches such as whole genome sequencing (WGS) and whole exome sequencing (WES).

Cancer personalized profiling (CAPP-Seq), tagged-amplicon (Tam-Seq), Safe-Seq, and targeted error correction (TEC-Seq) are some deep sequencing methods used for LB. CAPP-Seq uses population-based recurrent mutations for a given cancer and then quantifies the patient-specific mutations from ctDNA; it requires a minimum of 1 mL of plasma with at least 4 ng of input DNA to detect ctDNA at a VAF of 0.01% (Newman et al., 2014). Tam-Seq is an amplicon-based method whereby primers selectively amplify regions of interest before being tagged with barcodes in a subsequent PCR step, resulting in a VAF of 2%. This was then augmented with an enhanced Tam-Seq approach that captures a VAF of 0.02% with improved amplification of DNA and an optimized calling algorithm (Gale et al., 2018). Similar to Tam-Seq, Safe-Seq is an amplicon-based method that assigns a unique identifier to the template before the amplification step in order to improve the accuracy in detecting mutant variants (Kinde et al., 2011). Lastly, TEC-Seq is a method whereby a combination of barcodes are used to better discriminate true and false positive variants. Briefly, as in Safe-Seq, DNA fragments are assigned unique barcodes prior to amplification. In addition, the positions of paired-end fragments are used as barcodes to distinguish one molecule from another. In doing so, the sensitivity exceeds 97% with a VAF of 0.05% (Phallen et al., 2017; Bohers et al., 2021).

Untargeted approaches are less sensitive, more costly and depend on a more advanced skillset in utilizing the required technology; in return, they are ideal for discovery of new mutations and drug targets with a variety of genome-wide alterations. WES targets and sequences the coding regions of the genome, which is efficient but less sensitive than other methods since the exome represents less than 2% of the genome harbouring around 85% of known disease-related variants (van Dijk et al., 2014). It is sufficient in identifying novel mutations with an allele fraction over 5% or following disease progression and response to therapy in advanced cancers with high ctDNA burden (Bohers et al., 2021). In contrast, WGS comprehensively assesses the entire genome at high resolution, thereby identifying known and unknown single nucleotide variants in both coding and noncoding regions, insertions/deletions, CNVs and structural variants; it is limited by cost and the need for specialized skills to interpret large datasets.

#### 2.1.3 Epigenetic analyses

PCR and NGS can interrogate the presence of mutations and CNVs but cannot detect methylation changes that are implicated in tumorigenesis, which sometimes temporally precede the acquisition of single nucleotide variants. Initial whole genome bisulfite sequencing (WGBS) studies showed that interrogation of hundreds of thousands of CpG islands can provide useful information about DNA methylation markers (Corcoran and Chabner, 2018); however, this approach is limited by cost and the efficiency in yielding DNA after bisulfite conversion (Worm-Orntoft et al., 2017). Instead, quantitative whole-genome methylation assays that do not rely on bisulfite treatment, such as cell-free methylated DNA with immunoprecipitation (cfMeDIP-seq), can efficiently identify tumour specific methylation of CpG clusters in tumour suppressor genes from peripheral blood samples as small as 30 ng with enough sensitivity to: (1) detect cancer cells early on in disease (Chen et al., 2020) and (2) identify the origin of tumours in cancers of unknown primary (Moran et al., 2016; Gaitsch et al., 2023).

Newer technologies including the assessment of open chromatin with the assay for transposase-accessible chromatin sequencing (ATAC-seq) can be combined with NGS and PCR to yield optimally sensitive and specific epigenomic results (Buenrostro et al., 2015). However, methylation analyses generally require a relatively high sample input compared to genotyping applications, making them more challenging to adopt in minority cell-type investigations (Gaitsch et al., 2023), unless the number of markers per cell type is significantly increased (Loyfer et al., 2023).

### 2.2 CTCs

CTCs are intact cancer cells that are shed by the tumour into the bloodstream, either as single cells or in clusters. Most are highly differentiated, while some have stem cell-like properties (Wu and Chu, 2022). They have a half-life of 1–2.5 h before they are destroyed by immune cells. Some adapt and evade death by undergoing epithelial-to-mesenchymal transition allowing them to continue interacting with platelets and immune cells to promote cancer cell survival, proliferation and formation of metastases (Micalizzi et al., 2017; Gkountela et al., 2019). While they are released at all stages of tumorigenesis, they are particularly more abundant in advanced metastatic stage and as such, they can be a biomarker of both the presence of cancer and its stage (Yu et al., 2011). A recent analysis of 2,436 patients with stage IV breast cancer further showed that even amongst patients who have metastatic disease, patients having <5CTC/7.5 mL had significantly longer median overall survival than those with more CTCs (36.3 months versus 16.0 months, *p* < 0.0001), independent of the tumour molecular variables and location (Cristofallini et al., 2019).

#### 2.2.1 CTC enrichment technologies

**FIGURE 2:**
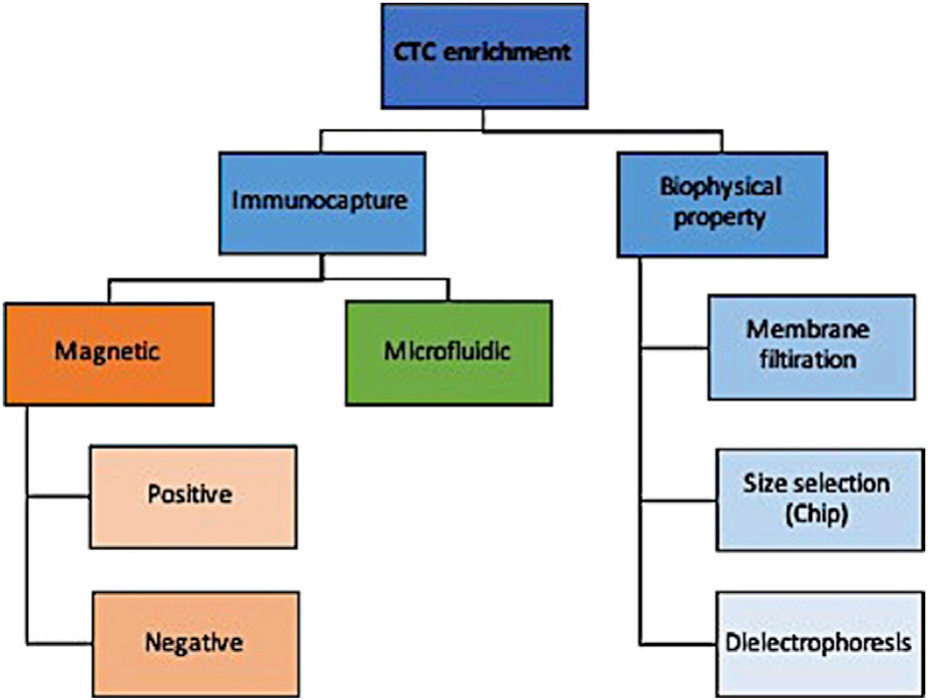
Schematic outlining the main CTC enrichment methods.

There are numerous technologies to enrich CTC from normal and diseased cells that can be broadly categorized into those that enrich based on surface cell markers and those that distinguish based on biophysical properties (Figure 2). While an exhaustive description of these technologies is outside the scope of this review and can be found by a paper by Rushton and others (Rushton et al., 2021), we briefly summarize here the main approaches and their indications.

Briefly, immunocapture methods use either magnetic or microfluidic properties to selectively enrich for target markers. Using magnetic properties, epithelial-based target markers on the surface of CTCs can be positively enriched or those present on leukocytes can be negatively enriched. Positive enrichment with anti-EpCAM antibodies is less useful for non-epithelial pathologies where epithelial-to-mesenchymal transition helps them evade detection (Hayes et al., 2006; Mikolajczyk et al., 2011). Negative enrichment technologies use magnetic beads to deplete leukocytes without selecting for EpCAM, albeit at lower recovery rates (Rushton et al., 2021).

An alternative to immunocapture is biophysical property selection. A simple example of that is membrane filtration whereby a pressure gradient allows for blood flows to flow over a filter that has pores of various sizes and shapes (Desitter et al., 2011; Kaifi et al., 2015); this has been applied to both live and fixed cells for a variety of cancers (Desitter et al., 2011; Nicolazzo et al., 2018). CTCs can also be separated from leukocytes by size and deformability using devices that have multiple gap sizes on a chip with higher detection rates reported than immunocapture methods such as CellSearch (Hosokawa et al., 2013). Their limitation, like membrane filtration methods, include low throughput and low specificity when dealing with small CTCs with similar sizes to leukocytes. Lastly, CTCs can be enriched by dielectrophoresis whereby cells pass over a field and the current pulls CTCs out of the stream with good reported recovery rates (55%–68%) and high viability (Le Du et al., 2020).

### 2.3 Exosomes/extracellular vesicles (EVs)

Exosomes or tumour derived extracellular vesicles (EVs) are small vesicles (30–120 nm in diameter) derived from the endosomal pathway that have a lipid bilayer and contain nucleic acids, proteins and lipids (Théry et al., 2018). Unlike cells, they cannot replicate. They play a critical role in cellular communication as they can secrete their contents and genomic message to other cells. Each type of cancer cell secretes its own specific EV with unique cargoes, making it an ideal biomarker to determine the presence, type and stage of cancer present (Tian et al., 2019). The diagnostic potential of microRNA and protein analyses of EV contents are increasingly being studied in various oncological and non-oncological diseases ranging from cardiovascular disease to organ transplantation (Zhou et al., 2020). Lastly, as a vesicle, it can be considered as a potential vehicle to deliver targeted drugs to certain cells (Liang et al., 2021).

EVs are potentially a superior source for liquid biopsy in that they are highly accessible, their lipid bilayer portends higher stability of the transported content (even at various storage temperatures (Cheruvanky et al., 2007)), and they have clear markers that can distinguish their cell type and content, making them ideal for diagnostics (Hoshino et al., 2015). Using EVs ultimately enhances liquid biopsy sensitivity and specificity in that: (1) there is more cfDNA to be captured in EVs than in the plasma, (2) the mutational frequency of exoDNA is even higher than cfDNA (Allenson et al., 2017; Mohrmann et al., 2018), and (3) combining the results of various EV contents can provide diagnostic information with greater accuracy (Madhavan et al., 2015).

**FIGURE 3:**
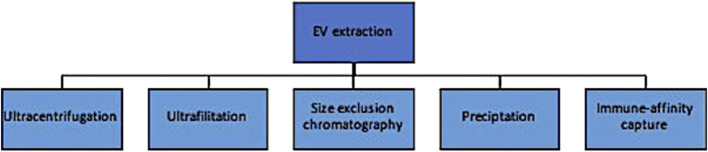
Schematic outlining the main EV extraction methods and their selection properties.

Traditional EV extraction methods are similar to CTC enrichment in that they rely primarily on biophysical and chemical properties and can produce results with heterogenous levels of yield and purity (Figure 3). These include ultracentrifugation, ultrafiltration and size exclusion chromatography, precipitation methods, and immune-affinity capture techniques. The most commonly used approach is differential ultracentrifugation, where EVs are isolated based on both size and density (Lishvits et al., 2015). Large volumes of biofluids can be processed at relatively low cost; however, there are several disadvantages of this method, including: (1) the suboptimal purity from its inability to distinguish particles with small differences in biophysical properties, and (2) the amount of labour needed to optimize the centrifuge to account for differences in sample volumes, density, temperature, etc. Ultrafiltration and size exclusion chromatography isolate exosomes by size through a series of pores in a filter or gel, respectively (OFagain et al., 2017). The latter is considered especially effective at respecting the exosome’s integrity in that particle flow is maintained by gravity alone without the use of chemical or pressure gradients. This is a simpler approach than differential ultracentrifugation and more cost effective, but complicated by lipoprotein contamination and loss of exosomes that inadvertently clog the filter pores. Precipitation techniques incubate exosomes with precipitants and then concentrate these aggregates in a buffer after low-speed centrifugation. Importantly, this method concentrates but does not separate EVs from other components, affecting its purity (Yakubovich et al., 2022). Lastly, immuno-affinity measures are similar to those described for CTC enrichment and increase the purity, at the expense of recovery (Thery et al., 2018; Zhou et al., 2020).

Evidently, the main issues with EV extraction methods surround biased results from either contamination of contents or low yield of the desired EV at the isolation step. Focusing on clinical potential, methods that prioritize purity are potentially more optimal for biomarker detection whereas those that preserve functional integrity might be more relevant in targeted treatment planning. Newer technologies are being investigated that attempt to overcome these shortcomings and are reviewed elsewhere (Yakubovich et al., 2022); nevertheless, the labour intensity, cost and heterogenous results preclude their widespread clinical adoption at this time.

## 3 Liquid biopsy techniques and applications for VM care

LBs have been largely applied in the field of oncology with growing support from health-regulated commercially available tools and technology. The genetic information gleaned reflects the spatial and temporal evolution of a tumour even at low frequencies, making LBs ideal for early detection of disease, treatment selection, survival prognostication, treatment response surveillance and identifying resistant colonies. Their ability to detect and monitor molecular footprints from a multitude of biofluids enables the expansion of LBs to non-oncological pathologies, including originally autoimmune disorders, and then neurodegenerative diseases, infectious diseases, prenatal screening, transplant medicine, and cardiovascular disorders, to name a few (Wu and Chu, 2022).

**FIGURE 4:**
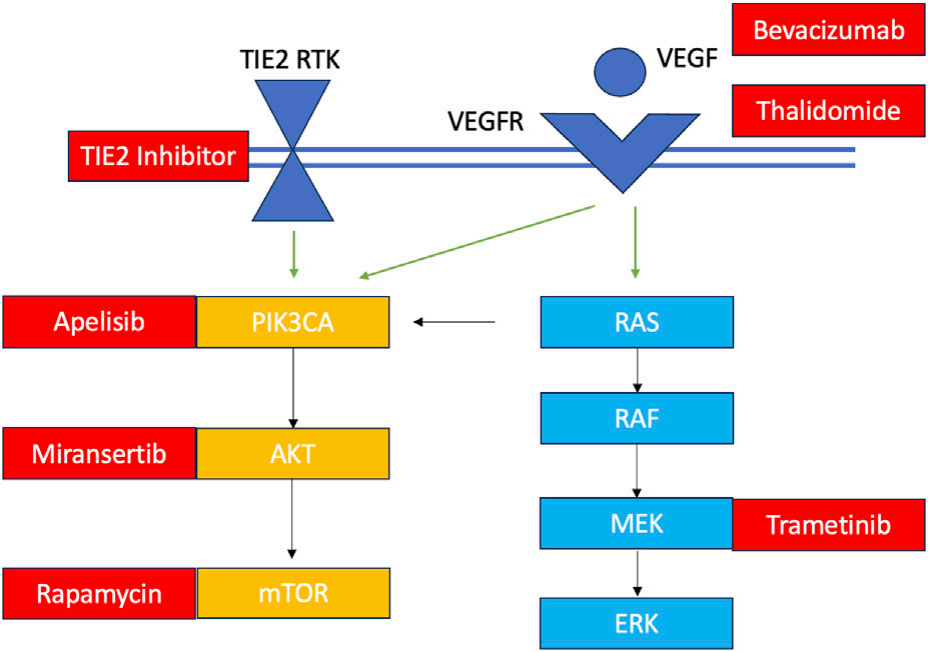
Overview of VM mutations and their targeted therapeutics TIE2 RTK: TEK gene receptor tyrosine kinase; VEGF, vascular endothelial growth factor; VEGFR, vascular endothelial growth factor receptor. Somatic TIE2 and PIK3CA mutations are observed in in venous malformations, lymphatic malformations and PROS. Some central lymphatic anomalies such as Gorham Stout Disease (GSD) and kaposiform lymphangiomatosis (KLA) harbour RAS mutations while patients with central conducting lymphatic anomalies (CCLA) have ARAF mutations. Arteriovenous malformations harbour KRAS and MAP mutations. Currently investigated targeted therapies for each component of the pathway are shown in red.

Their application to VM care is appealing given the relatively new discovery of their shared molecular development with various cancers and the potential to repurpose cancer drugs for their clinical management. Briefly, over the past 2 decades, scientists have learned that only about 5% of VMs are familial in nature, meaning they occur due to an inherited loss-of-function germline mutation with a somatic second hit (Mansur and Radovanovic, 2023). The remaining malformations are all sporadic with gain-of-function mutations occurring in a cluster of non-gametal cells that pertain to one of two major cellular signalling pathways: the PI3KCA-AKT-mTOR and the RAS-RAF-MEK-ERK pathways (Figure 4). These pathways play important roles in governing cellular survival, growth, proliferation, motility and cell death (Pan et al., 2021). Their implication in a variety of cancers has led to the approval of various small molecule inhibitors in oncology that are being explored through observational studies and clinical trials for VM pathologies (Figure 4) (Quiesser et al., 2021; Mansur and Radovanovic, 2023). Most of these investigations focused on palliative patients with extracranial vascular lesions, and only one investigation was held of a patient with an AVM of the central nervous system. Given their condition, a molecular diagnosis with an open biopsy was not deemed necessary in many cases, and patients were treated empirically with oral targeted therapeutics presuming they had the aberrant pathology within the pathway of interest. Clinical improvement was heterogenous, depending on the drug, dose and patient characteristics, ranging from 40%–80%, alongside radiological improvement noted in several clinical trials (Van Damme et al., 2020; Quiesser et al., 2021; Mansur and Radovanovic, 2023). From the current literature, it is evident that small molecule inhibitors can play a role in future VM care; however, it is unclear how and why some patients have a meaningful clinical response and others do not. Recapitulating the patient’s unique mutational burden is the first step in determining their candidacy for pathway-targeted therapy. Patients with VMs (especially those of the central nervous system), have lesions that may be particularly challenge to access and prone to bleeding; hence, a more minimally-invasive diagnostic method would be prudent to minimize risk.

A challenge in applying LBs to VM diagnostics is that somatic mutations in VMs occur in significantly smaller fractional abundances than in tumours; hence they are often below the level of detection with current technology from peripheral plasma draws. Instead, lesional blood that is immediately intimate to the VM would best capture the molecular aberrations without a tissue diagnosis. In order to explore this hypothesis, Palmieri and others were the first to conduct LBs of plasma from the efferent vein of five extra-cranial arteriovenous malformations (AVMs) during angiography where they were already intimate to the lesion for embolization, and as such, acquisition of blood as a biofluid did not portend an additional risk to the patient (Palmieri et al., 2020). They opted to use cfDNA as a biomarker; variant calling was performed using Torrent Suite and for variant annotation, Ion Reporter Software 5.10 (ThermoFisher Scientific) was used (Life Technologies, Carlsbad, CA 92008, United States of America). They were able to identify KRAS mutations (p.G12V and pG12D) in all specimens despite low variant allele frequencies (1.18%–4.19%). This group subsequently conducted several other studies using the same LB technique for VM patients: (1) in one study, they found novel mutations in the *MET* gene in 4/4 patients with extracranial severe venolymphatic malformations, with a VAF of 0.09%–0.97% (Palmieri et al., 2021a); (2) the same year, they published a case series of 7 patients with Klippel-Trenanay syndrome, all of whom harboured mutations in the *PIK3CA* gene found at a VAF of 0.18%–1.23% in both peripheral and efferent vein samples (albeit higher VAF from efferent vein) (Palmieri et al., 2021b); and (3) they later validated their findings in a group of 28 patients with various types of VMs and found mutant positive somatic mutations in the efferent vein of 75% patients with a VAF of 0.07%–6.64% (Serio et al., 2022).

This novel diagnostic approach was also employed by the Bennet lab in Seattle in eight AVMs, three venous malformations (VeM) and 7 lymphatic malformations (LMs) of the body. They captured LBs of AVMs and VeMs just prior to endovascular embolization and bleomycin therapies, respectively. Cyst fluid from LMs was acquired at bedside using ultrasound guidance. Most LBs were able to identify the corresponding somatic mutation using ddPCR; negative LBs were derived from either insufficient blood volumes or at a time point after VM treatment, including surgical resection (Zenner et al., 2021). In all studies, cfDNA LBs from the efferent vein of extracranial VMs were feasible and safe.

More recently, a different non-invasive biopsy approach was developed by Cooke’s group in San Francisco that doesn’t generate ctDNA from lesional blood, but rather uses endovascular means to perform an endoluminal biopsy of the endothelial wall of intracranial AVMs (Winkler et al., 2022). This technique is slightly more invasive and requires the deployment of an endovascular coil just prior to embolic treatment; the coil remains in intimate contact with the endothelial wall for a brief period of time and then is retrieved. DNA is isolated from the cells around the coil, endothelial cells were selected and then subjected to microarray for gene expression. They too found that this technique was successful in detecting mutations including KRAS in four patients without complication (Winkler et al., 2022).

These novel diagnostic techniques have significant clinical implications and require validation in a larger cohort of VMs and in patients with VM syndromes of unknown genetic origin. Given the lower abundance of circulating biomarkers in VMs compared to other diseases such as tumours, specific considerations need to be made when planning an LB application, starting from the selection of biofluid to the choice of sequencing technology. Here we highlight some of these considerations in light of the information reviewed from the existing literature in other disease entities, and more recently, the findings from these case series in VM populations.

### 3.1 Biofluid collection

The mutational load of VMs lie primarily in the endothelial cell, which then sheds into the local blood; hence, blood would be the most obvious choice of biomarker. Specifically, plasma is known to have a higher yield of biomarkers compared to serum and less chance of leukocyte contamination (Lee et al., 2020). Next, one must consider whether it is safe to procure a plasma sample that is intimate to the vascular lesion. This is often done at time of angiography (unless the lesion is superficial and can be accessed through direct puncture) and must balance the risk of sample collection with the necessity for this information. For example, an endoluminal biopsy might be relevant when a coil is already being deployed, but introducing a coil without the intention for embolization would put the patient at an increased risk. Similarly, we understand conceptually that the mutational load driving AVM development lies on the venous side and as such, we expect the efferent blood to be enriched in available biomarkers (Palmieri et al., 2020); however, if a transvenous approach is not already planned for the patient’s treatment, then gaining that access and deploying the required catheters would also introduce additional (and perhaps unnecessary) risks. These considerations are especially important in research studies that aim to validate techniques, where an ethical study would aim to minimize study risks for all participants.

If deemed safe to obtain a sample, the next question is what is the minimum level of detection necessary to render a positive result for the specific VM in question, and whether a sufficient plasma sample can be obtained to effectively extract sufficient genomic information to reach that sensitivity. For example, mutations in cfDNA might be more prevalent from lesional blood in extracranial VMs compared to intracranial VMs due to the heterogeneity found in endothelial biology in VMs across body sites (Waelchli et al., 2021). Many common cfDNA and EV isolation kits require a minimum of 1 mL of plasma, which in turn reflects around a minimum of 3–4 mL of whole blood. This might be challenging for some VMs such as intracranial AVMs with small caliber afferent and efferent vessels, or VeMs that have had recurrent sclerotherapy treatments with enhanced fibrotic tissue. In fact, Zenner’s group discussed how they found that majority of their patients who had negative liquid biopsies had insufficient plasma for optimal cfDNA extraction (Zenner et al., 2021). One option to improve upon the sensitivity of low volume samples is to increase the concentration of the sample or use a multiplexed approach to determine the presence of multiple genomic alterations that together support the presence of disease (Van der Pol and Mouliere, 2019).

Lastly, sample collection methods need to preserve precious biomarkers while avoiding contamination. This starts with careful handling of samples with a sterile approach. The choice of collection tube is known to have an effect on the biomarker yield. There are classic EDTA tubes and various long-term storage blood collection tubes (BCTs) that have preservatives to prevent cell lysis. Several studies compared the use of these tubes and found conflicting findings on which tube renders the highest yield and purity (Alidousty et al., 2017; Ward Ghalawat et al., 2019; Zhao et al., 2019; Zenner et al., 2021). Nevertheless, if using EDTA tubes, the blood must be spun within 4 h to optimize yield (Zhao et al., 2019). While the product monograph for some long-term BCTs such as Streck tubes recommend spinning the blood and isolating cfDNA within 14 days of collection, it has been found that yield is higher if done within the first couple of days after collection (Diaz et al., 2016).

### 3.2 Sample preparation

Once the blood is obtained safely in the desired collection tube, it is typically spun to separate the plasma from the buffy coat and red blood cells. Studies have shown that a two-step spin protocol is optimal for enhanced purity and yield of cfDNA whereas a single-step is optimal for recovery of cfRNA (Sorber et al., 2019; Fleischhacker and Schmidt, 2020). The first is a low-speed spin that removed blood cells and prevents cell lysis, that is then followed by a quick higher speed spin that removes unwanted debris (Sorber et al., 2019). Adding a third centrifugation step does not significantly change the cfDNA yield, and as such, is not typically conducted (Till et al., 2021).

### 3.3 Isolation and purification

A specific biomarker must be chosen before employing an appropriate isolation and purification method. While EVs have their advantages over cfDNA, the limitations already mentioned in their extraction methods make them less ideal for VM pathologies, which already pose challenges with biomarker yield and sensitivity. Instead, cfDNA would be a good biomarker to start with and can be combined with cfRNA for various markers of angiogenesis, proliferation and inflammation. Storage of either plasma or final cfDNA can be achieved at −80°C to optimize workflow in real-world situations; however, the goal would be to minimize freeze-thaw cycles to prevent cfDNA degradation.

As mentioned before, cfDNA kits differ in the volumes they can handle (automated for high throughput versus manual preparation) and the downstream application. Studies have shown than salting out methods might improve yield compared to silica-based membrane column methods (Seong et al., 2022.). Palmieri and others used the MagMax cell-free Total Nucleic Acid Isolation Kit (ThermoFisher Scientific, Waltham, Mass) in all of their studies, which relies on magnetic beads to isolate DNA as opposed to silica membrane methods; this might have supported the cfDNA yield observed from lesional blood (Palmieri et al., 2020; Palmieri et al., 2021a; Palmieri et al., 2021b; Serio et al., 2022).

### 3.4 Biomarker sequencing

The choice of sequencing technology depends on the type of VM tissue being explored, the range of VAF being detected, and the available resources to run the application. For VMs with known genetic mutations, ddPCR might be the most optimal technique in that it has the greatest sensitivity and is largely available at most centres. This might become relevant for VMs of the central nervous system, where the VAF might be below the level of detection of some NGS applications. Conversely, for unique cases of complex mixed VMs or VMs with widely unknown target mutations (i.e., dural arteriovenous fistulae), an exploratory approach with NGS would be preferred.

## 4 Conclusion

In the last few decades, the role of LBs has been increasingly explored and expanded upon with improvements in technologies for both biomarker detection and sequencing. Some of these are more highly validated for certain disease entities and are being adopted into clinical practice (Therry et al., 2018; Caputo et al., 2023). Our growing understanding of the molecular biology of vascular malformations is paving the way for personalized medicine in the treatment of these complex disorders. The necessity for molecular diagnosis will become imperative in stratifying patients to appropriate treatments. Future larger studies are needed to validate the appropriate biomarker selection and extraction method from precious samples that may be difficult to access, low in volume, and have a low abundance of biomarkers compared to other disease entities. More information is needed on specific pre-processing steps to optimize yield and purity specifically in VM biofluids, and the application of novel technologies specifically for the diagnostic question at hand. Ultimately, in order for LBs to permeate the clinical field of VM theragnostics, we must eventually see a standardization and automation of methods, from collection to storage and finally bioinformatic analyses, that yields consistent and cost-effective results for patients.

## References

[B1] Adashek J. J. Janku F. Kurzrock R. (2021). Signed in blood: circulating tumor DNA in cancer diagnosis, treatment and screening. Cancers 13 (14), 3600. 10.3390/cancers13143600 34298813 PMC8306582

[B2] Alidousty C. Brandes C. Heydt S. Wagener M. Wittersheim S. C. Schäfer B. (2017). Comparison of blood collection tubes from three different manufacturers for the collection of cell-free DNA for liquid biopsy mutation testing. J. Mol. Diagn 19, 801–804. 10.1016/j.jmoldx.2017.06.004 28732213

[B3] Allenson K. Castillo J. San Lucas F. A. Scelo G. Kim D. U. Bernard V. (2017). High prevalence of mutant KRAS in circulating exosome-derived DNA from early-stage pancreatic cancer patients. Ann. Oncol. 28 (4), 741–747. 10.1093/annonc/mdx004 28104621 PMC5834026

[B4] Bielo L. B. Trapani D. Repetto M. Crimini E. Valenza C. Belli C. (2023). Variant allele frequency: a decision-making tool in precision oncology? Trends Cancer 9 (12), 1058–1068. 10.1016/j.trecan.2023.08.011 37704501

[B5] Bohers E. Viailly P. J. Jardin F. (2021). cfDNA sequencing: technological approaches and bioinformatic issues. Pharm. (Basel) 14 (6), 596. 10.3390/ph14060596 PMC8234829 34205827

[B6] Buenrostro J. D. Wu B. Chang H. Y. Greenleaf W. J. (2015). ATAC-seq: a method for assaying chromatin accessibility genome-wide. Curr. Protoc. Mol. Biol. 109, 21–29. 10.1002/0471142727.mb2129s109 PMC4374986 25559105

[B7] Caputo V. Ciardiello F. Della Corte C. M. Martini G. Troiani T. Napolitano S. (2023). Diagnostic value of liquid biopsy in the era of precision medicine: 10 years of clinical evidence in cancer. Explor Target Antitumor Ther. 4, 102–138. 10.37349/etat.2023.00125 36937316 PMC10017193

[B8] Chen X. Gole J. Gore A. He Q. Lu M. Min J. (2020). Non-invasive early detection of cancer four years before conventional diagnosis using a blood test. Nat. Commun. 11, 1–10. 10.1038/s41467-020-17316-z 32694610 PMC7374162

[B9] Cheruvanky A. Zhou H. Pisitkun T. Kopp J. B. Knepper M. A. Yuen P. S. (2007). Rapid isolation of urinary exosomal biomarkers using a nanomembrane ultrafiltration concentrator. Am. J. Physiol. Ren. Physiol. 292 (5), F1657–F1661. 10.1152/ajprenal.00434.2006 PMC2271070 17229675

[B10] Corcoran R. B. Chabner B. A. (2018). Application of cell-free DNA analysis to cancer treatment. N. Engl. J. Med. 379, 1754–1765. 10.1056/NEJMra1706174 30380390

[B11] Couto J. A. Huang A. Y. Konczyk D. J. Goss J. A. Fishman S. J. Mulliken J. B. (2017). Somatic MAP21 mutations are associated with extracranial arteriovenous malformation. Am. J. Hum. Genet. 100 (3), 546–554. 10.1016/j.ajhg.2017.01.018 28190454 PMC5339083

[B12] Cristofanilli M. Pierga J. Y. Reuben J. Rademaker A. Davis A. A. Peeters D. J. (2019). The clinical use of circulating tumor cells (CTCs) enumeration for staging of metastatic breast cancer (MBC): international expert consensus paper. Crit. Rev. Oncol. 134, 39–45. 10.1016/j.critrevonc.2018.12.004 30771872

[B13] Derdeyn C. P. Zipfel G. J. Albuquerque F. C. Cooke D. L. Feldmann E. Sheehan J. P. (2017). Management of brain arteriovenous malformations: a scientific statement for healthcare professionals from the American Heart Association/American Stroke Association. Stroke 48, e200–e224. 10.1161/STR.0000000000000134 28642352

[B14] Desitter I. Guerrouahen B. S. Benali-Furet N. Wechsler J. Jänne P. A. Kuang Y. (2011). A new device for rapid isolation by size and characterization of rare circulating tumor cells. Anticancer Res. 31, 427–441. PMID: 21378321. 21378321

[B15] Diaz I. M. Nocon A. Mehnert D. H. Fredebohm J. Diehl F. Holtrup F. (2016). Performance of Streck cfDNA blood collection tubes for liquid biopsy testing. PLoS ONE 11, e0166354. 10.1371/journal.pone.0166354 27832189 PMC5104415

[B16] Diaz L. A. Jr Bardelli A. (2014). Liquid biopsies: genotyping circulating tumor DNA. J. Clin. Oncol. 32, 579–586. 10.1200/JCO.2012.45.2011 24449238 PMC4820760

[B17] Diehl F. Schmidt K. Choti M. A. Romans K. Goodman S. Li M. (2008). Circulating mutant DNA to assess tumor dynamics. Nat. Med. 14, 985–990. 10.1038/nm.1789 18670422 PMC2820391

[B18] Di Sario G. Rossella V. Famulari E. S. Maurizio A. Lazarevic D. Giannese F. (2023). Enhancing clinical potential of liquid biopsy through a multi-omic approach: a systematic review. Front. Genet. 14, 1152470. 10.3389/fgene.2023.1152470 37077538 PMC10109350

[B19] Dressman D. Yan H. Traverso G. Kinzler K. W. Vogelstein B. (2003). Transforming single DNA molecules into fluorescent magnetic particles for detection and enumeration of genetic variations. Proc. Natl. Acad. Sci. USA. 100, 8817–8822. 10.1073/pnas.1133470100 12857956 PMC166396

[B20] Esposito A. Criscitiello C. Trapani D. Curigliano G. (2017). The emerging role of “liquid biopsies,” circulating tumor cells, and circulating cell-free tumor DNA in lung cancer diagnosis and identification of resistance mutations. Curr. Oncol. Rep. 19 (1), 1. 10.1007/s11912-017-0564-y 28110461

[B21] Fish J. E. Flores Suarez C. P. Boudreau E. Herman A. M. Gutierrez M. C. Gustafson D. (2020). Somatic gain of KRAS function in the endothelium is sufficient to cause vascular malformations that require MEK but not PI3K signaling. Circulation Res. 127 (6), 727–743. 10.1161/CIRCRESAHA.119.316500 32552404 PMC7447191

[B22] Fleischhacker M. Schmidt B. (2020). Pre-analytical issues in liquid biopsy – where do we stand? J. Laboratory Med. 44 (3), 117–142. 10.1515/labmed-2019-0167

[B23] Gaitsch H. Franklin R. J. M. Reich D. S. (2023). Cell-free DNA-based liquid biopsies in neurology. Brain 146 (5), 1758–1774. 10.1093/brain/awac438 36408894 PMC10151188

[B24] Gale D. Lawson A. R. J. Howarth K. Madi M. Durham B. Smalley S. (2018). Development of a highly sensitive liquid biopsy platform to detect clinically-relevant cancer mutations at low allele fractions in cell-free DNA. PLoS ONE 13, e0194630. 10.1371/journal.pone.0194630 29547634 PMC5856404

[B25] Gkountela S. Castro-Giner F. Szczerba B. M. Vetter M. Landin J. Scherrer R. (2019). Circulating tumor cell clustering shapes DNA methylation to enable metastasis seeding. Cell 176, 98–112. 10.1016/j.cell.2018.11.046 30633912 PMC6363966

[B26] Gross B. A. Du R. (2013). Natural history of cerebral arteriovenous malformations: a meta-analysis. J. Neurosurg. 118, 437–443. 10.3171/2012.10.JNS121280 23198804

[B27] Hayes D. F. Cristofanilli M. Budd G. T. Ellis M. J. Stopeck A. Miller M. C. (2006). Circulating tumor cells at each follow-up time point during therapy of metastatic breast cancer patients predict progression-free and overall survival. Clin. Cancer Res. 12, 4218–4224. 10.1038/s41523-021-00281-1 16857794

[B28] Heitzer E. Haque I. S. Roberts C. E. S. Speicher M. R. (2019). Current and future perspectives of liquid biopsies in genomics-driven oncology. Nat. Rev. Genet. 20, 71–88. 10.1038/s41576-018-0071-5 30410101

[B29] Hong T. Yan Y. Li J. Radovanovic I. Ma X. Shao Y. W. (2019). High prevalence of KRAS/BRAF somatic mutations in brain and spinal cord arteriovenous malformations. Brain J. Neurol. 142 (1), 23–34. 10.1093/brain/awy307 30544177

[B30] Hoshino A. Costa-Silva B. Shen T. L. Rodrigues G. Hashimoto A. Tesic Mark M. (2015). Tumour exosome integrins determine organotropic metastasis. Nature 527, 329–335. 10.1038/nature15756 26524530 PMC4788391

[B31] Hosokawa M. Kenmotsu H. Koh Y. Yoshino T. Yoshikawa T. Naito T. (2013). Size-based isolation of circulating tumor cells in lung cancer patients using a microcavity array system. PLoS ONE 8, e67466. 10.1371/journal.pone.0067466 23840710 PMC3696066

[B32] Kaifi J. T. Kunkel M. Das A. Harouaka R. A. Dicker D. T. Li G. (2015). Circulating tumor cell isolation during resection of colorectal cancer lung and liver metastases: a prospective trial with different detection techniques. Cancer Biol. Ther. 16, 699–708. 10.1080/15384047.2015.1030556 25807199 PMC4622016

[B33] Kinde I. Wu J. Papadopoulos N. Kinzler K. W. Vogelstein B. (2011). Detection and quantification of rare mutations with massively parallel sequencing. Proc. Natl. Acad. Sci. U. S. A. 108 (23), 9530–9535. 10.1073/pnas.110.5422108 21586637 PMC3111315

[B34] Kunimoto K. Yamamoto Y. Jinnin M. (2022). Classification of vascular anomalies and molecular biology. Int. J. Mol. Sci. 23, 2358. 10.3390/ijms23042358 35216474 PMC8876303

[B35] Lamy P. J. Van Der Leest P. Lozano N. Becht C. Duboeuf F. Groen H. J. M. (2020). Mass spectrometry as a highly sensitive method for specific circulating tumor DNA analysis in nsclc: a comparison study. Cancers 12, 3002. 10.3390/molecules24244423 33081150 PMC7602843

[B36] Le Du F. Fujii T. Kida K. Davis D. W. Park M. Liu D. D. (2020). EpCAM-independent isolation of circulating tumor cells with epithelial-to-mesenchymal transition and cancer stem cell phenotypes using ApoStream(R) in patients with breast cancer treated with primary systemic therapy. PLoS ONE 15, e0229903. 10.1371/journal.pone.0229903 32214335 PMC7098555

[B37] Lee J. S. Kim M. Seong M. W. Kim H. S. Lee Y. K. Kang H. J. (2020). Plasma vs. serum in circulating tumor DNA measurement: characterization by DNA fragment sizing and digital droplet polymerase chain reaction. Clin. Chem. Lab. Med. 58 (4), 527–532. 10.1515/cclm-2019-0896 31874093

[B38] Liang Y. Duan L. Lu J. Xia J. (2021). Engineering exosomes for targeted drug delivery. Theranostics 11, 3183–3195. 10.7150/thno.52570 33537081 PMC7847680

[B39] Lishvits M. A. Khomyakova E. Evtushenko E. G. Lazarev V. N. Kulemin N. A. Semina S. E. (2015). Isolation of exosomes by differential centrifugation: theoretical analysis of a commonly used protocol. Sci. Rep. 5, 17319. 10.1038/srep17319 26616523 PMC4663484

[B40] Loyfer N. Magenheim J. Peretz A. Cann G. Bredno J. Klochendler A. (2023). A DNA methylation atlas of normal human cell types. Nature 613 (7943), 355–364. 10.1038/s41586-022-05580-6 36599988 PMC9811898

[B41] Madhavan B. Yue S. Galli U. Rana S. Gross W. Müller M. (2015). Combined evaluation of a panel of protein and miRNA serum-exosome biomarkers for pancreatic cancer diagnosis increases sensitivity and specificity. Int. J. Cancer 136 (11), 2616–2627. 10.1002/ijc.29324 25388097

[B42] Mansur A. Kostynskyy A. Krings T. Agid R. Radovanovic I. Mendes Pereira V. (2021). The safety profile and angioarchitectural changes after acute targeted embolization of ruptured arteriovenous malformations. J. Neurosurg. 135 (6), 1598–1607. 10.3171/2020.9.JNS201558 33962377

[B43] Mansur A. Radovanovic I. (2023). Vascular malformations: an overview of their molecular pathways, detection of mutational profiles and subsequent targets for drug therapy. Front. Neurol. 14, 1099328. 10.3389/fneur.2023.1099328 36846125 PMC9950274

[B44] McCafferty I. (2015). Management of low-flow vascular malformations: clinical presentation patient selection, imaging and treatment. Cardiovasc Interv. Radiol. 38, 1082–1104. 10.1007/s00270-015-1085-4 25895482

[B45] Micalizzi D. S. Maheswaran S. Haber D. A. (2017). A conduit to metastasis: circulating tumor cell biology. Genes Dev. 31, 1827–1840. 10.1101/gad.305805.117 29051388 PMC5695084

[B46] Mikolajczyk S. D. Millar L. S. Tsinberg P. Coutts S. M. Zomorrodi M. Pham T. (2011). Detection of EpCAM-negative and cytokeratin-negative circulating tumor cells in peripheral blood. J. Oncol. 2011, 252361. 10.1155/2011/252361 21577258 PMC3090615

[B47] Möhrmann L. Huang H. J. Hong D. S. Tsimberidou A. M. Fu S. Piha-Paul S. A. (2018). Liquid biopsies using plasma exosomal nucleic acids and plasma cell-free DNA compared with clinical outcomes of patients with advanced cancers. Clin. Cancer Res. 24 (1), 181–188. 10.1158/1078-0432.CCR-17-2007 29051321 PMC5754253

[B48] Moran S. Martínez-Cardús A. Sayols S. Musulén E. Balañá C. Estival-Gonzalez A. (2016). Epigenetic profiling to classify cancer of unknown primary: a multicentre, retrospective analysis. Lancet Oncol. 17 (10), 1386–1395. 10.1016/S1470-2045(16)30297-2 27575023

[B49] Moss J. Magenheim J. Neiman D. Zemmour H. Loyfer N. Korach A. (2018). Comprehensive human cell-type methylation atlas reveals origins of circulating cell-free DNA in health and disease. Nat. Commun. 9 (1), 5068. 10.1038/s41467-018-07466-6 30498206 PMC6265251

[B50] Newman A. M. Bratman S. V. To J. Wynne J. F. Eclov N. C. (2014). An ultrasensitive method for quantifying circulating tumor DNA with broad patient coverage. Nat. Med. 20 (5), 548–554. 10.1038/nm.3519 24705333 PMC4016134

[B51] Nicolazzo C. Colangelo L. Corsi A. Carpino G. Gradilone A. Sonato C. (2018). Biopsy in rare cancers: lessons from hemangiopericytoma. Anal. Cell. Pathol. 2018, 9718585. 10.1155/2018/9718585 PMC5863319 29707475

[B52] Nikanjam M. Kato S. Kurzrock R. (2022). Liquid biopsy: current technology and clinical applications. J. Hematol Oncol 15, 131. 10.1186/s13045-022-01351-y 36096847 PMC9465933

[B53] Nikolaev S. I. Vetiska S. Bonilla X. Boudreau E. Jauhiainen S. Jahromi B. R. (2018). Somatic activating KRAS mutations in arteriovenous malformations of the brain. N. Engl. J. Med. 378, 250–261. 10.1056/NEJMoa1709449 29298116 PMC8161530

[B54] Ofagain C. Cummins P. M. O'Connor B. F. (2017). Gel-filtration chromatography. Methods Mol. Biol. 1485, 15–25. 10.1007/978-1-4939-6412-3_2 27730546 PMC7121854

[B55] Palmieri M. Currò A. Tommasi A. Di Sarno L. Doddato G. Baldassarri M. (2020). Cell-free DNA next-generation sequencing liquid biopsy as a new revolutionary approach for arteriovenous malformation. JVS Vasc. Sci. 1, 176–180. 10.1016/j.jvssci.2020.08.002 34617046 PMC8489236

[B56] Palmieri M. Di Sarno L. Tommasi A. (2021). MET somatic activation mutations are responsible for lymphovenous malformation and can be identified using cell-free DNA next generation sequencing liquid biopsy. J. Vasc. Surg. Venous Lymphat. Disord. 9 (3), 740–744. 10.1016/j.jvsv.2020.07.015 32858245

[B57] Palmieri M. Pinto A. M. Di Blasio L. Currò A. Monica V. Sarno L. D. (2021). A pilot study of next generation sequencing-liquid biopsy on cell-free DNA as a no*vel non*-invasive diagnostic tool for Klippel-Trenaunay syndrome. Vascular 29 (1), 85–91. 10.1177/1708538120936421 32588787

[B58] Pan P. Weinsheimer S. Cooke D. Winkler E. Abla A. Kim H. (2021). Review of treatment and therapeutic targets in brain arteriovenous malformation. J. Cereb. Blood Flow. Metab. 41 (12), 3141–3156. 10.1177/0271678X211026771 34162280 PMC8669284

[B59] Phallen J. Sausen M. Adleff V. Leal A. Hruban C. White J. (2017). Direct detection of early-stage cancers using circulating tumor DNA. Sci. Transl. Med. 9, eaan2415. 10.1126/scitranslmed.aan2415 28814544 PMC6714979

[B60] Pyrak E. Krajczewski J. Kowalik A. Kudelski A. Jaworska A. (2019). Surface enhanced Raman spectroscopy for DNA biosensors— how far are we? Molecular 24, 4423. 10.3390/molecules24244423 PMC6943648 31817059

[B61] Quiesser A. Boon L. M. Vikkula M. (2018). Etiology and genetics of congenital vascular lesions. Otolaryngol. Clin. North Am. 51, 41–53. 10.1016/j.otc.2017.09.006 29217067

[B62] Quiesser A. Seront E. Boon L. M. Vikkula M. (2021). Genetic basis and therapies for vascular malformations. Circ. Res. 129, 155–173. 10.1161/CIRCRESAHA.121.318145 34166070

[B63] Raufi A. G. May M. S. Hadfield M. J. Seyhan A. A. El-Deiry W. S. (2023). Advances in liquid biopsy technology and implications for pancreatic cancer. Int. Mol. Sci. 24, 4238. 10.3390/ijms24044238 PMC9958987 36835649

[B64] Rushton A. J. Nteliopoulos G. Shaw J. A. Coombes R. C. (2021). A review of circulating tumour cell enrichment technologies. Cancers 139, 970. 10.3390/cancers13050970 PMC7956528 33652649

[B65] Seong H. Park J. Bae M. Shin S. (2022). Rapid and efficient extraction of cell-free DNA using homobifunctional crosslinkers. Biomedicines 10 (8), 1883. 10.3390/biomedicines10081883 36009429 PMC9405790

[B66] Serio V. B. Palmieri M. Loberti L. Granata S. Fallerini C. Vaghi M. (2022). Nosological and theranostic approach to vascular malformation through cfDNA NGS liquid biopsy. J. Clin. Med. 11 (13), 3740. 10.3390/jcm11133740 35807022 PMC9267326

[B67] Siravegna G. Bardelli A. (2014). Genotyping cell-free tumor DNA in the blood to detect residual disease and drug resistance. Genome Biol. 15, 449. 10.1186/s13059-014-0449-4 25222559 PMC4281953

[B68] Sorber L. Zwaenepoel K. Deschoolmeester V. Van Schil P. E. Van Meerbeeck J. Lardon F. (2017). Circulating cell-free nucleic acids and platelets as a liquid biopsy in the provision of personalized therapy for lung cancer patients. Lung Cancer 107, 100–107. 10.1016/j.lungcan.2016.04.026 27180141

[B69] Sorber L. Zwaenepoel K. Jacobs J. De Winne K. Goethals S. Reclusa P. (2019). Circulating cell-free DNA and RNA analysis as liquid biopsy: optimal centrifugation protocol. Cancers 11, 458. 10.3390/cancers11040458 30935089 PMC6521186

[B70] Stroun M. Maurice P. Vasioukhin V. Lyautey J. Lederrey C. Lefort F. (2000). The origin and mechanism of circulating DNA. Ann. N. Y. Acad. Sci. 906, 161–168. 10.1111/j.1749-6632.2000.tb06608.x 10818614

[B71] Théry C. Witwer K. W. Aikawa E. Alcaraz M. J. Anderson J. D. Andriantsitohaina R. (2018). Minimal information for studies of extracellular vesicles 2018 (MISEV2018): a position statement of the International Society for Extracellular Vesicles and update of the MISEV2014 guidelines. J. Extracell. Vesicles 7, 1535750. 10.1080/20013078.2018.1535750 30637094 PMC6322352

[B72] Tian X. Shen H. Li Z. Wang T. Wang S. (2019). Tumor-derived exosomes, myeloid-derived suppressor cells, and tumor microenvironment. J. Hematol. Oncol. 12, 84. 10.1186/s13045-019-0772-z 31438991 PMC6704713

[B73] Till J. E. Black T. A. Gentile C. Abdalla A. Wang Z. Sangha H. K. (2021). Optimization of sources of circulating cell-free DNA variability for downstream molecular analysis. J. Mol. Diagn 23 (11), 1545–1552. 10.1016/j.jmoldx.2021.08.007 34454115 PMC8647427

[B74] Van Damme A. Seront E. Dekeuleneer V. Boon L. M. Vikkula M. (2020). New and emerging targeted therapies for vascular malformations. Am. J. Clin. Dermatol 21, 657–668. 10.1007/s40257-020-00528-w 32557381

[B75] Van der Pol Y. Mouliere F. (2019). Toward the early detection of cancer by decoding the epigenetic and environmental fingerprints of cell-free DNA. Cancer Cell 36, 350–368. 10.1016/j.ccell.2019.09.003 31614115

[B76] van Dijk E. L. Auger H. Jaszczyszyn Y. Thermes C. (2014). Ten years of next-generation sequencing technology. Trends Genet. 30, 418–426. 10.1016/j.tig.2014.07.001 25108476

[B77] Waelchli T. Ghobrial M. Schwab M. (2021). Molecular atlas of the human brain vasculature across development, adulthood and disease at the single-cell level. Nature. 10.1101/2021.10.18.464715 PMC11324530 38987604

[B78] Ward Gahlawat A. Lenhardt J. Witte T. Keitel D. Kaufhold A. Maass K. K. (2019). Evaluation of storage tubes for combined analysis of circulating nucleic acids in liquid biopsies. Int. J. Mol. Sci. 20 (3), 704. 10.3390/ijms20030704 30736351 PMC6387045

[B79] Wassef M. Blei F. A. Adams D. Alomari A. Baselga E. Berenstein A. (2015). Vascular anomalies classification: recommendations from the international society for the study of vascular anomalies. Pediatrics 136, e203–e214. 10.1542/peds.2014-3673 26055853

[B80] Winkler E. A. Wu D. Gil E. McCoy D. Narsinh K. Sun Z. (2022). Endoluminal biopsy for molecular profiling of human brain vascular malformations. Neurology 98, e1637–e1647. 10.1212/WNL.0000000000200109 35145012 PMC9052570

[B81] Worm Ørntoft M. B. Jensen S. Ø. Hansen T. B. Bramsen J. B. Andersen C. L. (2017). Comparative analysis of 12 different kits for bisulfite conversion of circulating cell-free DNA. Epigenetics 12 (8), 626–636. 10.1080/15592294.2017.1334024 28557629 PMC5687322

[B82] Wu H. J. Chu P. Y. (2022). Current and developing liquid biopsy techniques for breast cancer. Cancers 14, 2052. 10.3390/cancers14092052 35565189 PMC9105073

[B83] Yakubovich E. I. Polischouk A. G. Evtushenko V. I. (2022). Principles and problems of exosome isolation from biological fluids. Biochem. Mosc. Suppl. Ser. A Membr. Cell Biol. 16(2), 2115–126. 10.1134/S1990747822030096 PMC9202659 35730027

[B84] Yu M. Stott S. Toner M. Maheswaran S. Haber D. A. (2011). Circulating tumor cells: approaches to isolation and characterization. J. Cell Biol. 192, 373–382. 10.1083/jcb.201010021 21300848 PMC3101098

[B85] Zenner K. Jensen D. M. Cook T. T. Dmyterko V. Bly R. A. Ganti S. (2021). Cell-free DNA as a diagnostic analyte for molecular diagnosis of vascular malformations. Genet. Med. 23 (1), 123–130. 10.1038/s41436-020-00943-8 32884133 PMC7796969

[B86] Zhao Y. Li Y. Chen P. Li S. Luo J. Xia H. (2019). Performance comparison of blood collection tubes as liquid biopsy storage system for minimizing cfDNA contamination from genomic DNA. J. Clin. Lab. Anal. 33 (2), e22670. 10.1002/jcla.22670 30191594 PMC6818589

[B87] Zhou B. Xu K. Zheng X. Chen T. Wang J. Song Y. (2020). Application of exosomes as liquid biopsy in clinical diagnosis. Signal Transduct. Target Ther. 5 (1), 144. 10.1038/s41392-020-00258-9 32747657 PMC7400738

[B88] Zill O. A. Banks K. Fairclough S. R. Mortimer S. A. Vowles J. V. Mokhtari R. (2018). The landscape of actionable genomic alterations in cell-free circulating tumor DNA from 21,807 advanced cancer patients. Clin. Cancer Res. 24, 3528–3538. 10.1158/1078-0432.CCR-17-3837 29776953

